# Predictors of return to work for people on sick leave with depression, anxiety and stress: secondary analysis from a randomized controlled trial

**DOI:** 10.1007/s00420-023-01968-7

**Published:** 2023-03-18

**Authors:** Siv-Therese Bjørkedal, Jonas Fisker, Lone Christina Hellström, Andreas Hoff, Rie Mandrup Poulsen, Carsten Hjorthøj, Anders Bo Bojesen, Nicole Gremaud Rosenberg, Lene Falgaard Eplov

**Affiliations:** 1 Copenhagen Research Institute for Mental Health [CORE], Mental Health Services Capital Region of Denmark, University of Copenhagen, Gentofte Hospitalsvej 15.4, 2900 Hellerup, Denmark; 2 Mental Health Centre Copenhagen, Mental Health Services Capital Region of Denmark, 2200 Copenhagen, Denmark; 3 National Board of Social Services in Denmark, Edisonsvej 1, 5000 Odense, Denmark

**Keywords:** Occupational rehabilitation, Work participation, Sickness absence, Mental health, Prognostic factors

## Abstract

**Purpose:**

Knowledge about predictors of return to work (RTW) in people on sick leave with common mental disorders (CMDs) may inform the development of effective vocational rehabilitation interventions for this target group. In this study, we investigated predictors of RTW at 6 and 12 months in people on sick leave with depression, anxiety disorders or stress-related disorders.

**Methods:**

We have performed a secondary analysis, utilizing data from two RCTs that evaluated the efficacy of an integrated health care and vocational rehabilitation intervention. Data were obtained from mental health assessments, questionnaires and registers. Using Cox regression analysis, the relationship between baseline variables and RTW was analysed at 6 and 12 months after randomization within the group of CMD as a whole and within the subgroups of depression, anxiety and stress-related disorders.

**Results:**

Symptom burden and employment status at baseline predicted RTW in the CMD group (*n* = 1245) and in the three diagnostic subgroups at both time points. RTW self-efficacy predicted RTW in the depression group but not in the anxiety or stress subgroups.

**Conclusion:**

Many predictors of RTW were similar over time and, to some extent, across the CMD subgroups. Findings highlight the need not only to take health-related and psychological factors into account when developing vocational rehabilitation interventions but also to consider workplace strategies and options for support.

**Supplementary Information:**

The online version contains supplementary material available at 10.1007/s00420-023-01968-7.

## Introduction

Vocational rehabilitation encompasses goal-directed interventions with the core objective of enabling work participation (Waddell et al. 2008). Within mental health, recipients of vocational rehabilitation interventions are commonly divided into two groups: (a) people diagnosed with severe mental illness (SMI) (Frederick and VanderWeele 2019; Kinoshita et al. 2013; Modini et al. 2016) and (b) people diagnosed with common mental disorders (CMDs) (Mikkelsen and Rosholm 2018; Nigatu et al. 2016). Multiple systematic reviews have found that supported employment, often delivered as Individual Support and Placement (IPS), is effective for people with SMI (Bond et al. 2020; Crowther et al. 2001; Frederick and VanderWeele 2019; Kinoshita et al. 2013; Modini et al. 2016). Yet, this does not apply to people with CMD to the same extent (de Winter et al. 2022; Hellström et al. 2021). In a systematic review and meta-analysis from 2022, de Winter et al. found IPS to be more effective in populations with SMI than in populations with CMD (de Winter et al. 2022). In their meta-analysis, Hellström et al. concluded that studies have been unable to establish an effect of IPS on employment in people with major depression (Hellström et al. 2021). A systematic review of Return-to-Work (RTW) interventions for individuals on sick leave due to CMD found that interventions combining cognitive behavioural therapy, problem solving therapy and workplace change (e.g. reduced working hours) could reduce sickness absence with 13–30 days, corresponding to an effect size of *d* = 0.14. Although economically important, the effect size was small and perhaps not clinically relevant (Nigatu et al. 2016).

Knowledge about predictors of RTW can inform intervention development and subsequently improve vocational outcomes (de Vries et al. 2018; Kent et al. 2020). Factors predicting RTW among people with CMD encompass psychological factors, such as self-efficacy (Brenninkmeijer et al. 2019; Lagerveld et al. 2017; Volker et al. 2015), work expectance (Nielsen et al. 2012) and readiness to change (Hellström et al. 2022), as well as health-related factors, such as symptom severity and psychiatric comorbidity (de Winter et al. 2022; Hellström et al. 2022). Psychological and health-related factors are somewhat modifiable and commonly targeted in RTW interventions, for instance, through cognitive behavioural therapy (Joyce et al. 2016; Poulsen et al. 2017b). Findings from systematic reviews focusing on people on sick leave with CMD (Cornelius et al. 2011; Fisker et al. 2022) and depression (Ervasti et al. 2017; Lagerveld et al. 2010b; Volker et al. 2015) have shown that job position, labour market attachment and sociodemographic factors, such as age, civic status and education also predict RTW. These predictors indicate the existence of structural barriers to RTW that are not easily modifiable through vocational rehabilitation interventions (Hellström et al. 2022).

CMD is an umbrella term for a heterogeneous group of mental health conditions, such as depression, anxiety disorders and stress-related disorders (Hoedeman 2012; Poulsen et al. 2017a, b). Although these conditions have overlapping symptoms, their distinct features may impact RTW differently. In a cohort study, Mattila-Holappa et al. found that employees on sick leave with depression were less likely to return to work than those absent with stress-related disorders. Results also showed that older age (> 50 years) decreased the likelihood of RTW in the depression group but not in the stress group (Mattila-Holappa et al. 2017). Hence, factors associated with RTW in one condition may be overlooked when investigating the CMD group as a whole. To this date, systematic reviews of RTW interventions have investigated CMD as one group (Cornelius et al. 2011; Fisker et al. 2022; Nigatu et al. 2017) or focused on depression (Ervasti et al. 2017; Lagerveld et al. 2010b; Volker et al. 2015). In a systematic review by Fisker from 2022 (Fisker et al. 2022), none of the included studies investigated anxiety disorders separately. Instead, they looked at groups with anxiety and depression (Lammerts et al. 2017) or anxiety and stress (Kausto et al. 2017). Hence, we need more knowledge about factors predicting RTW in specific diagnostic subgroups—especially anxiety and stress-related disorders—in order to design customized vocational rehabilitation interventions for these target groups.

When it comes to vocational outcomes, longitudinal studies have demonstrated a profound variability in the RTW process among people with CMD (Hellström et al. 2018; Horn et al. 2022; Øyeflaten et al. 2012; Pedersen et al. 2016). A Dutch study showed that employees with burn-out and depressive disorders had slower RTW than employees with adjustment disorders and that increased age decreased the likelihood of RTW (Horn et al. 2022). In a Swedish cohort study, being on sick leave or receiving work disability pension 13 months after a sickness episode was predicted by being male, being unemployed and having only elementary education (Farrants et al. 2018). Hellström et al. showed that higher levels of functioning and readiness to change were associated with more rapid RTW (> 3 months) following sick leave with anxiety and depression (Hellström et al. 2018). Fisker et al. found that, among people absent from work with CMD, RTW after three months or less was associated with RTW expectations, while RTW after at least one year was associated with higher age and lower educational level (Fisker et al. 2022). These studies suggest that the trajectory of the RTW process is predicted not only by diagnosis but also by health-related, psychological and sociodemographic factors. Identifying factors that predict vocational outcomes at different time points may facilitate the development of effective RTW interventions and direct attention to subgroups of recipients who require customized interventions (Craig et al. 2008; Kent et al. 2020).

In order to develop effective vocational rehabilitation interventions tailored to specific conditions and groups at high risk of long-term sickness absence, we need more knowledge about what facilitates or hinders RTW over time. Thus, the objective of this study was to investigate predictors of RTW in people on sick leave with a CMD at specific time points; in the CMD group as a whole and in the subgroups hereafter referred to as the depression, anxiety and stress groups.

## Design and procedure

This study was designed as a prospective cohort study, using data obtained from two randomized controlled trials (RCT) in the Danish IBBIS project conducted between April 2016 and April 2018. In Danish, IBBIS is an acronym for “Integrated Health Care and Vocational Rehabilitation for Sick Leave Benefit Recipients”. The IBBIS project evaluated an integrated mental health care and vocational rehabilitation intervention for people on sick leave with depression, anxiety disorders or stress-related disorders. Trial participants were randomized to one of three intervention groups: (a) integrated vocational rehabilitation and mental health care; (b) mental health care alone and vocational rehabilitation as usual or (c) vocational rehabilitation and mental health care as usual. The primary outcome in both trials was time to RTW at 12 months (Hoff et al. 2022a, b; Poulsen et al. 2017a, b).

Participants were referred from jobcenters in the four Danish municipalities participating in the project (Copenhagen, Lyngby-Taarbæk, Gladsaxe and Gentofte). In Denmark, municipal jobcenters deliver public employment services, including sick leave benefits. The case manager handling the sick leave case could refer citizens to the IBBIS project if the cause of sick leave was suspected mental health problems. Citizens on sick leave from employment and from unemployment could participate in the project. If the citizen consented to participate in the IBBIS project, he or she was invited for a mental health assessment to determine eligibility for the RCT. Prior to the mental health assessment, participants filled out an online self-report questionnaire covering symptoms, functioning and various psychological aspects (e.g. self-efficacy and quality of life) (Poulsen et al. 2017a, 2017b). The mental health assessment was based on the MINI neuropsychiatric interview (Sheehan et al. 1998), which is a short, structured psychiatric interview performed by psychiatrists, psychologists, nurses and social workers trained in using the instrument. At the mental health assessment, participants were screened for ADHD symptoms, using the Adult ADHD Self-Report Scale version 1.1. (ASRS) Symptom Checklist (Kessler et al. 2005) and for indication of Personality Disorder, using the Standardized Assessment of Personality Abbreviated Scale (SAPAS) (Moran et al. 2003). We screened for ADHD symptoms and personality disorders because we theorized that such disorders could affect the management and outcome of the IBBIS intervention. If the mental health assessment indicated a need for acute help or treatment in secondary mental health care, the participant was excluded from the study and referred to relevant care. Individuals with dementia, substance or alcohol abuse, or an unstable medical condition could not participate in the IBBIS project.

A detailed description of the RCTs is published in two protocol papers (Poulsen et al. 2017a, b), and findings from the trials are reported in two papers by Hoff et al. (Hoff et al. 2022a, b).

In this study, the CMD group is regarded as a single unit but also divided into three subgroups: (1) a stress group comprising distress, adjustment disorders and exhaustion disorders; (2) an anxiety group comprising panic disorders, general anxiety disorders and social anxiety disorders; and (3) a depression group comprising mild, moderate and severe depression.

### Outcome

The outcome of interest in this study was time to stable RTW at 6 and 12 months after randomization in the IBBIS project, hereafter referred to as baseline. Stable RTW was defined as not receiving sick leave benefits for a consecutive period of four weeks. Data on sick leave benefits were obtained from the national DREAM database and the Income Statistics Register. The DREAM database is administered by the Danish Agency for Labor Market and Recruitment and includes all persons with a Danish civil personal registration number (CPR) who have received social benefits, or any other type of transfer income, on a weekly basis (Hjollund et al. 2007). The Income Statistics Register provides statistics on the Danish population’s income and tax deductions and contains individual-level data that can be linked to data from the DREAM database (The Income Statistics Register 2022).

### Predictor variables

#### Demographic variables

Demographic variables consisted of sex, age, education level, marital status and municipality affiliation. Data on these variables were obtained from The Danish Population Register and the DREAM database.

#### Health-related variables

Information about diagnosis and psychiatric comorbidity was obtained through the mental health assessment. Self-reported symptoms were measured using the Beck Depression Inventory (BDI II), a 21-item questionnaire assessing the intensity of depression (Beck et al. 1996); the 21-item Beck Anxiety Inventory (BAI) (Fydrich et al. 1992); the 10-item Cohens Perceived Stress Scale (PSS) (Lee 2012); the 50-item Four-Dimensional Symptom Questionnaire (4DSQ) that assesses common psychological symptoms of distress, depression, anxiety and somatization as separate dimensions, using four scores to indicate symptom level (Terluin et al. 2006), and the 26-item Karolinska Exhaustion Scale (KES) that evaluates the degree of exhaustion disorder (Saboonchi et al. 2013). Functioning was measured using the 5-item Work and Social Adjustment Scale (WSAS) that assesses functional impairment within work, home management, leisure activities and social relationships (Mundt et al. 2002). Symptoms of ADHD were detected using the ASRS v1.1, a self-report form based on the 18 DSM criteria. The instrument consists of two parts: a section A with 6 questions and a section B with 12 questions. Each question yields a score between 0 and 4. A person screens positive on the ASRS v1.1 if he or she answers yes to four or more of the Part A questions (Kessler et al. 2005). In this study, we adopted a pragmatic approach. Thus, participants’ ASRS scores were converted into a dichotomous variable: (a) ASRS score below 7, suggesting no ADHD symptoms, and (b) ASRS score of 7 or more, suggesting presence of ADHD symptoms.

#### Psychological variables

Quality of life was measured using the 16-item Flanagan’s Quality of Life Scale (QOLS) that includes five domains of quality of life (Burckhardt and Anderson 2003); self-efficacy regarding symptom management was assessed using the 18-item Illness Perception Questionnaire (IPQ); self-efficacy regarding RTW was measured using the 11-item Return-to-Work Self-Efficacy Scale (RTW-SE) (Lagerveld et al. 2010a) and general self-efficacy (optimistic beliefs about one’s ability to cope with a variety of difficult demands in life) was assessed using the 10-item General Self-Efficacy Scale (GSE) (Schwarzer and Jerusalem 1995).

At the mental health assessment, information about personality traits and indication of personality disorder were obtained with SAPAS, a brief interview consisting of eight dichotomously rated questions. All eight questions are derived from the Standardized Assessment of Personality and correspond to descriptive statements about the person, for instance, “do you have difficulties in finding and keeping friends?” or “do you depend on others a lot?” Answers can be scored 0 (absent) or 1 (present), and the sum generates an overall score between 0 and 8. In a validation study of SAPAS, Moran et al. found that when using a cut-off score of 3, sensitivity was 0.94 and specificity 0.85. The positive and negative predictive values of SAPAS were 0.89 and 0.92, respectively (Moran et al. 2003). In our study, participants’ scores on each of the SAPAS questions made up eight dichotomous variables. Overall SAPAS scores were converted into a dichotomous variable with a cut-off at 3 or more vs. below 3, indicating presence or absence of personality disorder.

#### Work-related variables

Work-related variables included sick leave from employment or unemployment at baseline, duration of sick leave episode at baseline and socioeconomic position (salaried manager or self-employed, salaried worker, student or unemployed (receiving transfer income)). Information on work-related variables was obtained from the DREAM database and the Income Statistics Register.

### Statistical analysis

Baseline values were calculated for the CMD group and for the stress, anxiety and depression subgroups. Continuous variables were presented with mean and standard deviations (SD) and categorical variables with count (*n*) and percentages. Pairwise Pearson correlations were calculated for all baseline variables to assess multicollinearity (supplementary material). Correlation estimates for baseline variables were between 0.3 and 0.7. For each of the four groups (CMD, stress, anxiety and depression), Cox regression analysis was used to calculate the relation between baseline variables and time to RTW at 6 and 12 months, measured as hazard ratios (HRs). All potential predictor variables were analysed in two consecutive steps. First, univariable analyses of each predictor–outcome relationship were conducted for all potential predictors without any adjustments. Next, variables associated with the outcome with *p* values < 0.10 were included the subsequent multivariable analysis. The multicollinearity analysis showed that some predictors measured the same construct, e.g. BAI and 4DSQ Anxiety. In cases where both were significant in the univariable analysis we chose only the one with the lowest missingness for the multivariable analysis. In the multivariable analysis, backward stepwise elimination of predictors was used with a 5% significance criterion. We had to take into account that two-thirds of the population had received an intervention (an integrated mental health and vocational rehabilitation intervention or a mental health intervention as an adjunct to usual case management in the jobcenters). Therefore, all analyses were adjusted for treatment allocation group (1/2/3). Every multivariable analysis was carried out using multiple imputation of all missing values. Predictive mean matching in chained equations (Van Buuren and Groothuis-Oudshoorn 2011) based on all observed baseline data was used to generate 250 imputation sets. Multiple imputation estimation was carried out for the multiple Cox regressions (single-predictor Cox regression estimates reflect only complete cases). A stepwise backward elimination of predictors was implemented for multiple imputations by accepting only those predictors that were kept in at least 80% of the stepwise elimination procedures for the 250 iterations. Data were analysed using R version 3.6.1. Backward selection was carried out using the “pec” package (Mogensen et al. 2012).

## Results

A total of 1245 participants were included in the CMD group. Among the subgroups, the stress group had the largest sample size (*n* = 636), followed by the depression group (*n* = 387) and the anxiety group (*n* = 222). Participants’ mean age was 43.3 years (SD: 10.5), and 24.9% were male. Missing values were found in self-report questionnaires and information gathered at the mental health assessments. For the CMD group, missing values were found in 32% of the cases. This means that missing values were found in information obtained at the mental health assessments and/or in the self-report questionnaires for 32% of the participants. In the stress group, missing values were found in 24.7% of the cases, in the depression group in 28.1% and in the anxiety group in 7.7%. The missing values were mainly ascribed to SAPAS, ASRS, KES and QOLS. All baseline characteristics, including the range of missing values for each analysed group, are shown in Table 1.

**Table 1:** Sample characteristics at baseline, overall, as common mental disorders group, and distributed by subgroups, anxiety, depression and stress-related disorders

	Common mental disorders (*n* = 865–1245^a^)	Stress (*n* = 479–636^a^)	Anxiety (*n* = 202–222^a^)	Depression (*n* = 278–387^a^)
Demographic characteristics				
Age - (range^b^ 21–64) mean (SD)	43.25 (10.47)	44.57(9.95)	40.7 (10.75)	42.56 (10.83)
Sex (male), *n* (%)	310 (24.9)	149 (23.4)	62 (27.9)	99 (25.6)
Status-married, *n* (%)	634 (50.9)	337 (53)	116 (52.3)	181 (46.8)
Education level				
Primary education, *n* (%)	381 (30.6)	170 (26.7)	78 (35.1)	133 (34.4)
Secondary or vocational, *n* (%)	472 (37.9)	236 (37.1)	82 (36.9)	154 (39.8)
Professional or academic degree, *n* (%)	392 (31.5)	230 (36.2)	62 (27.9)	100 (25.8)
Municipality				
Copenhagen (%)	757 (60.8)	381 (59.9)	133 (59.9)	243 (62.8)
Gentofte (%)	132 (10.6)	81 (12.7)	17 (7.7)	34 (8.8)
Gladsaxe (%)	193 (15.5)	87 (13.7)	34 (15.3)	72 (18.6)
Lyngby (%)	163 (13.1)	87 (13.7)	38 (17.1)	38 (9.8)
Health-related characteristics				
Symptoms				
The Beck depression inventory (range 0–54) mean (SD)	24.1 (9.6)	20.6 (8.5)	22.9 (9.1)	30.4 (8.5)
Becks anxiety inventory (range 0–54) mean (SD)	18.5 (9.2)	15.3 (8)	21.7 (9)	22 (9.3)
Cohen’s perceived stress scale^c^ (range 5–40) mean (SD)	24.4 (5.6)	23 (5.5)	24.4 (5.3)	26.6 (5)
The four-dimensional symptom questionnaire-anxiety (range 0–24) mean (SD)	5.9 (5.4)	4 (3.9)	7.8 (5.9)	7.9 (6)
The four-dimensional symptom questionnaire-depression (range 0–12) mean (SD)	3 (3.1)	2 (2.4)	2.7 (3)	4.9 (3.4)
The four-dimensional symptom questionnaire-distress (range 1–32) mean (SD)	19.7 (6.7)	17.4 (6.2)	18.9 (6.6)	23.8 (5.7)
The four-dimensional symptom questionnaire-somatization (range 0–32) mean (SD)	12.6 (6.7)	11 (6.3)	13.7 (6.6)	14.6 (6.8)
The Karolinska exhaustion scale (range 37–122) mean (SD)^c^	83.1 (13.6)	79.6 (13.2)	82.4 (13.8)	89.4 (11.8)
Functioning				
The work and social adjustment scale^2^ (range 0–40) mean (SD)	23.6 (7.9)	21.5 (7.9)	22.7 (7.5)	27.6 (6.5)
Comorbidity				
Comorbidity: (yes %)	247 (19.8)	52 (8.2)	54 (24.3)	141 (36.4)
Adult ADHD self-report scale total score (≥ 7), *n* (%)	551 (49.7)	266 (45.9)	124 (62.9)	161 (48.5)
Psychological factors				
Quality of life				
Flanagans quality of life scale^2^ (range 15–98) mean (SD)	61.5 (13.4)	65.6 (12)	62.2 (11.7)	54.2 (13.4)
Self-efficacy				
The illness perception questionnaire (range 0–24) mean (SD)	15.1 (3.6)	15.7 (3.5)	15.3 (3.5)	13.9 (3.6)
Return-to-work self-efficacy (range 0–44) mean (SD)	13.3 (7.1)	14.2 (7.3)	14.3 (7)	11.5 (6.5)
The general self-efficacy scale^2^ (range 10–40) mean (SD)	23.5 (6.4)	25.1 (6.1)	22.9 (6)	21.1 (6.4)
Personality				
SAPAS-difficulty making/keeping friends (Yes), *n* (%)	140 (12.1)	46 (7.7)	38 (18.5)	56 (15.8)
SAPAS-describe yourself as a loner (Yes), *n* (%)	160 (13.8)	59 (9.8)	35 (17)	66 (18.6)
SAPAS-trust other people (yes), *n* (%)	1024 (88.3)	547 (91.5)	172 (83.9)	305 (85.4)
SAPAS-lose temper easily (yes), *n* (%)	93 (8)	42 (7)	21 (10.2)	30 (8.5)
SAPAS-an impulsive person (yes), *n* (%)	369 (31.8)	193 (32.2)	60 (29.3)	116 (32.7)
SAPAS-a worrier (Yes), *n* (%)	551 (47.6)	244 (40.6)	146 (72.3)	161 (45.4)
SAPAS-depend lot on others (yes), *n* (%)	127 (10.9)	50 (8.3)	34 (16.5)	43 (12.1)
SAPAS-a perfectionist (yes), *n* (%)	608 (52.9)	296 (49.7)	114 (57.3)	198 (55.6)
SAPAS total (≥ 3), *n* (%)	594 (53.9)	275 (47.9)	133 (68.6)	186 (55.5)
Work-related factors				
Labour market: employed (yes), *n* (%)	1014 (81.4)	542 (85.2)	172 (77.5)	300 (77.5)
Socio-economic status				
Salaried manager or self-employed, *n* (%)	358 (28.8)	216 (34.0)	60 (27)	82 (21.2)
Salaried worker, *n* (%)	374 (30.0)	180 (28.3)	60 (27)	134 (34.6)
Income transfer, *n* (%)	133 (10.7)	70 (11.0)	15 (6.8)	48 (12.4)
In education, *n* (%)	380 (30.5)	170 (26.7)	87 (39.2)	123 (31.8)
Sick leave duration at randomization				
Sick leave duration-weeks (range 0–53) mean (SD)	10.7 (3.8)	10.6 (3.5)	10.8 (4.6)	10.7 (3.8)

^a^Due to missing cases, *n* varies

^b^Age range as determined by the common mental disorder group

^c^Range of scale measures are determined by the actual minimum and maximum values in the common mental disorder group and not by the scale range

Tables 2 and 3 present results for the CMD and stress groups from the univariate and multivariate analyses at 6 and 12 months, while Tables 4 and 5 show results for the anxiety and depression groups. Below is a summary of factors predicting slower RTW at 6 and 12 months in the four groups.

**Table 2:** Demographic, health-related, psychological and work-related predictors for RTW in the CMD group

Predictor variable	6 Months		12 Months	
	Univariate	Multivariate	Univariate	Multivariate
	HR (95% CI)	HR (95% CI)	HR (95% CI)	HR (95% CI)
Demographic variables				
Age^1^	1.08 (1.01–1.17)*		1.06 (0.99–1.13)	
Sex				
Female	Ref		Ref	Ref
Male	0.84 (0.70–1.02)		0.80 (0.68–0.94)**	0.76 (0.65–0.90)**
Marital status				
Not married	Ref		Ref	Ref
Married	1.24 (1.06–1.46)**		1.28 (1.12–1.47)***	1.21 (1.06–1.39)**
Educational level				
Primary education only	Ref		Ref	
Secondary or vocational	1.33 (1.09–1.63)**		1.18 (1.00–1.41)	
Professional or academic degree	1.60 (1.31–1.97)***		1.52 (1.28–1.81)***	
Municipality				
Copenhagen	Ref		Ref	
Gentofte	1.04 (0.80–1.36)		1.11 (0.88–1.38)	
Gladsaxe	1.12 (0.90–1.40)		1.17 (0.96–1.41)	
Lyngby	1.49 (1.19–1.86)***		1.38 (1.13–1.68)**	
Health-related variables				
Symptoms				
The Beck depression inventory^2^	0.98 (0.97–0.98)***		0.98 (0.97–0.99)***	
Becks anxiety inventory^2^	0.98 (0.97–0.98)***		0.98 (0.97–0.99)***	
Cohens perceived stress scale^2^	0.96 (0.95–0.97)***		0.97 (0.96–0.98)***	
The four-dimensional symptom questionnaire-anxiety^2^	0.96 (0.94–0.97)***		0.96 (0.95–0.98)***	
The four-dimensional symptom questionnaire-depression^2^	0.93 (0.90–0.96)***		0.94 (0.92–0.97)***	
The four-dimensional symptom questionnaire-distress^2^	0.97 (0.95–0.98)***		0.97 (0.96–0.98)***	
The four-dimensional symptom questionnaire-somatization^2^	0.98 (0.97–0.99)***		0.98 (0.97–0.99)***	
The Karolinska exhaustion scale^2^	0.98 (0.98–0.99)***		0.99 (0.98–0.99)***	
Functioning				
The work and social adjustment scale^2^	0.97 (0.96–0.98)***		0.97 (0.96–0.98)***	
Diagnosis and comorbidity				
Diagnosis				
Anxiety	Ref		Ref	
Depression	0.76 (0.59–0.97)*		0.79 (0.64–0.97)*	
Stress	1.24 (1.00–1.55)*		1.20 (1.00–1.45)	
Comorbidity				
No	Ref		Ref	Ref
Yes	0.64 (0.51–0.80)***		0.66 (0.55–0.80)***	0.76 (0.63–0.92)**
Adult ADHD self-report scale (ASRS) ≥ 7 vs < 7				
ASRS < 7	Ref		Ref	
ASRS ≥ 7	0.85 (0.72–1.00)		0.87 (0.76–1.01)	
Psychological variables				
Quality of life				
Flanagans quality of life scale^2^	1.02 (1.02–1.03)***	1.01 (1.01–1.02)***	1.02 (1.01–1.02)***	1.01 (1.00–1.02)**
Self-efficacy				
The illness perception questionnaire^2^	1.02 (1.00–1.05)*		1.03 (1.01–1.05)**	
Return-to-work self-efficacy^2^	1.04 (1.03–1.05)***	1.03 (1.02–1.05)***	1.03 (1.02–1.04)***	1.03 (1.01–1.04)***
The general self-efficacy scale^2^	1.03 (1.02–1.05)***		1.03 (1.01–1.04)***	
Personality				
SAPAS-difficulty making/keeping friends				
No	Ref	Ref	Ref	
Yes	0.54 (0.40–0.73)***	0.66 (0.49–0.90)**	0.64 (0.50–0.81)***	
SAPAS-describe yourself as a loner				
No	Ref		Ref	
Yes	0.78 (0.61–1.01)		0.73 (0.59–0.92)**	
SAPAS-trust other people				
Yes	Ref		Ref	
No	1.24 (0.95–1.61)		1.23 (0.98–1.54)	
SAPAS-lose temper easily				
No	Ref		Ref	
Yes	0.76 (0.54–1.06)		0.81 (0.62–1.07)	
SAPAS-an impulsive person				
No	Ref		Ref	
Yes	0.87 (0.72–1.03)		0.90 (0.77–1.05)	
SAPAS-a worrier				
No	Ref		Ref	
Yes	0.88 (0.75–1.04)		0.93 (0.81–1.08)	
SAPAS-depend lot on others				
No	Ref		Ref	
Yes	0.90 (0.68–1.17)		0.85 (0.67–1.07)	
SAPAS-a perfectionist				
No	Ref		Ref	
Yes	0.93 (0.79–1.10)		0.94 (0.82–1.08)	
SAPAS total (> 3)				
SAPAS total < 3	Ref		Ref	
SAPAS ≥ 3	0.81 (0.68–0.95)*		0.85 (0.74–0.99)*	
Work-related variables				
On sick leave from unemployment or employment at randomization				
Unemployment	Ref	Ref	Ref	Ref
Employment	3.50 (2.61–4.68) ***	3.36 (2.50–4.52) ***	2.73 (2.19–3.39) ***	2.64 (2.11–3.29) ***
Socioeconomic position				
Salaried manager or self-employed	Ref		Ref	
Salaried worker	0.75 (0.61–0.91)**		0.81 (0.68–0.96)*	
Income transfer	0.58 (0.43–0.79)***		0.59 (0.45–0.77)***	
In education	0.77 (0.63–0.94)*		0.82 (0.69–0.98)*	
Sick leave duration at randomization				
Sick leave duration at randomization^3^	1.00 (0.98–1.02)		0.99 (0.98–1.01)	

Cox proportional hazards model with HR < 1 indicating a longer time to return to work and HR > 1 indicating a shorter return to work

*Significant at < 0.05

**Significant at < 0.01

***Significant at < 0.001

^1^10-year increase

^2^1-point increase

^3^1-week increase

**Table 3:** Demographic, health-related, psychological and work-related predictors for RTW in the stress group

Predictor variable	6 Months		12 Months	
	Univariate	Multivariate	Univariate	Multivariate
	HR (95% CI)	HR (95% CI)	HR (95% CI)	HR (95% CI)
Demographic variables				
Age^1^	0.99 (0.89–1.10)		0.99 (0.90–1.09)	
Sex				
Female	Ref		Ref	
Male	0.84 (0.65–1.08)		0.83 (0.66–1.05)	
Marital status				
Not married	Ref		Ref	
Married	I.10 (0.89–1.36)		1.11 (0.93–1.34)	
Educational level				
Primary education only	Ref		Ref	
Secondary or vocational	1.48 (1.12–1.96)**		1.26 (0.99–1.60)	
Professional or academic degree	1.55 (1.17–2.04)**		1.45 (1.14–1.84)**	
Municipality:				
Copenhagen (ref)	Ref		Ref	
Gentofte	0.86 (0.62–1.21)		0.89 (0.67–1.19)	
Gladsaxe	1.17 (0.86–1.58)		1.25 (0.96–1.63)	
Lyngby	1.30 (0.97–1.74)		1.16 (0.88–1.52)	
Health-related variables				
Symptoms				
The Beck depression inventory^2^	0.98 (0.97–0.99)**		0.98 (0.97–0.99)***	
Becks anxiety inventory^2^	0.98 (0.97–0.99)**		0.98 (0.97–0.99)***	
Cohen’s perceived stress scale^2^	0.98 (0.96–1.00)*		0.98 (0.96–0.99)**	
The four-dimensional symptom questionnaire-anxiety^2^	0.98 (0.95–1.01)		0.97 (0.95–1.00)*	
The four-dimensional symptom questionnaire-depression^2^	0.94 (0.90–0.99)**		0.95 (0.92–0.99)*	
The four-dimensional symptom questionnaire-distress^2^	0.98 (0.96–0.99)**		0.98 (0.96–0.99)**	
The four-dimensional symptom questionnaire-somatization^2^	0.98 (0.96–1.00)*		0.98 (0.96–0.99)**	
The Karolinska exhaustion scale^2^	0.99 (0.98–0.99)***		0.99 (0.98–0.99)***	
Functioning				
The work and social adjustment scale^2^	0.98 (0.96–0.99)***		0.98 (0.97–0.99)***	
Diagnosis and comorbidity				
Diagnosis				
Anxiety	N/A		N/A	
Depression	N/A		N/A	
Stress	N/A		N/A	
Comorbidity				
No	Ref		Ref	
Yes	0.64 (0.51–0.80)***		0.45 (0.29–0.68)***	0.44 (0.28–0.67)***
Adult ADHD self-report scale (ASRS) ≥ 7 vs < 7				
ASRS < 7	Ref		Ref	
ASRS ≥ 7	0.88 (0.71–1.10)		0.88 (0.72–1.07)	
Psychological variables				
Quality of life				
Flanagans quality of life scale^2^	1.02 (1.01–1.03)***		1.02 (1.01–1.03)***	
Self-efficacy				
The illness perception questionnaire^2^	1.02 (0.99–1.05)		1.02 (0.99–1.04)	
Return-to-work self-efficacy^2^	1.02 (1.01–1.04)***		1.02 (1.01–1.03)***	
The general self-efficacy scale^2^	1.03 (1.01–1.04)**		1.03 (1.01–1.04)***	
Personality				
SAPAS-difficulty making/keeping friends				
No	Ref		Ref	
Yes	0.56 (0.34–0.91)*		0.59 (0.39–0.89)*	
SAPAS-describe yourself as a loner				
No	Ref		Ref	
Yes	1.04 (0.73–1.49)		0.92 (0.66–1.29)	
SAPAS-trust other people				
Yes	Ref		Ref	
No	0.88 (0.61–1.26)		0.93 (0.67–1.30)	
SAPAS-lose temper easily				
No	Ref		Ref	
Yes	0.72 (0.45–1.15)		0.83 (0.57–1.22)	
SAPAS-an impulsive person				
No	Ref		Ref	
Yes	0.81 (0.64–1.03)		0.81 (0.65–1.00)*	
SAPAS-a worrier				
No	Ref		Ref	
Yes	0.90 (0.73–1.12)		0.95 (0.78–1.15)	
SAPAS-depend lot on others				
No	Ref		Ref	
Yes	0.91 (0.61–1.36)		0.83 (0.58–1.20)	
SAPAS-a perfectionist				
No	Ref		Ref	
Yes	1.20 (0.96–1.48)		1.18 (0.97–1.43)	
SAPAS total (≥ 3)				
SAPAS total < 3	Ref		Ref	
SAPAS total ≥ 3	0.90 (0.72–1.11)		0.92 (0.76–1.12)	
Work-related variables				
On sick leave from unemployment or employment at randomization				
Unemployment	Ref	Ref	Ref	Ref
Employment	4.59 (2.86–7.38)***	4.59 (2.85–7.39)***	3.63 (2.54–5.18)***	3.66 (2.56–5.23)***
Socioeconomic status:				
Salaried manager or self-employed	Ref		Ref	
Salaried worker	0.79 (0.60–1.02)		0.82 (0.65–1.04)	
Income transfer	0.68 (0.46–1.01)		0.57 (0.40–0.82) **	
In education	0.98 (0.75–1.26)		0.99 (0.79–1.25)	
Sick leave duration at randomization				
Sick leave duration at randomization^3^	1.02 (0.99–1.04)		1.01 (0.99–1.04)	

Cox proportional hazards model with HR < 1 indicating a longer time to return to work and HR > 1 indicating a shorter return to work

*Significant at < 0.05

**Significant at < 0.01

***Significant at < 0.001

^1^10-year increase

^2^1-point increase

^3^1-week increase

**Table 4:** Demographic, health-related, psychological and work-related predictors for RTW in the anxiety group

Predicting variables	6 Months		12 Months	
	Univariate	Multivariate	Univariate	Multivariate
	HR (95% CI)	HR (95% CI)	HR (95% CI)	HR (95% CI)
Demographic variables				
Age^1^	1.11 (0.93–1.33)		1.07 (0.92–1.24)	
Sex				
Female	Ref		Ref	
Male	0.81 (0.52–1.27)		0.76 (0.52–1.12)	
Marital status				
Not married	Ref		Ref	
Married	1.41 (0.95–2.09)		1.15 (0.83–1.61)	
Educational level				
Primary education only	Ref		Ref	
Secondary or vocational	1.41 (0.89–2.25)		1.43 (0.96–2.13)	
Professional or academic degree	1.53 (0.94–2.50)		1.59 (1.05–2.42)*	
Municipality				
Copenhagen	Ref		Ref	
Gentofte	1.12 (0.53–2.35)		1.01 (0.54–1.89)	
Gladsaxe	1.25 (0.73–2.14)		1.11 (0.69–1.77)	
Lyngby	1.61 (0.99–2.61)		1.35 (0.88–2.08)	
Health-related variables				
Symptoms				
The Beck depression inventory^2^	0.98 (0.96–1.00)		0.99 (0.97–1.01)	
Becks anxiety inventory^2^	0.98 (0.96–1.00)		0.99 (0.97–1.01)	
Cohen’s Perceived Stress Scale^2^	0.96 (0.92–0.99) *		0.97 (0.94–1.00)*	
The four-dimensional symptom questionnaire-anxiety^2^	0.95 (0.92–0.99)**		0.97 (0.94–1.00)*	
The four-dimensional symptom questionnaire-depression^2^	0.96 (0.89–1.02)		0.96 (0.90–1.01)	
The four-dimensional symptom questionnaire-distress^2^	0.97 (0.94–1.00)*		0.96 (0.94–0.99)**	
The four-dimensional symptom questionnaire-somatization^2^	0.98 (0.95–1.01)		0.98 (0.95–1.00)	
The Karolinska exhaustion scale^2^	0.99 (0.98–1.00)		0.99 (0.98–1.01)	
Functioning				
The work and social adjustment scale^2^	0.98 (0.96–1.01)		0.98 (0.96–1.01)	
Diagnosis and comorbidity				
Diagnosis:				
Anxiety	N/A		N/A	
Depression	N/A		N/A	
Stress	N/A		N/A	
Comorbidity				
No	Ref		Ref	
Yes	0.73 (0.46–1.19)		0.95 (0.65–1.38)	
Adult ADHD self-report scale (ASRS) ≥ 7 vs < 7				
ASRS < 7	Ref		Ref	
ASRS ≥ 7	0.96 (0.63–1.45)		1.04 (0.73–1.48)	
Psychological variables				
Quality of life				
Flanagans quality of life scale^2^	1.02 (1.01–1.04)**		1.02 (1.01–1.04)**	1.02 (1.00–1.03)*
Self-efficacy				
The illness perception questionnaire^2^	0.99 (0.94–1.05)		1.00 (0.95–1.05)	
Return-to-work self-efficay^2^	1.02 (0.99–1.05)		1.01 (0.98–1.03)	
The general self-efficacy scale^2^	1.03 (1.00–1.06)*		1.02 (1.00–1.05)	
Personality				
SAPAS-difficulty making/keeping friends				
No	Ref		Ref	
Yes	0.91 (0.55–1.51)		0.83 (0.53–1.30)	
SAPAS-describe yourself as a loner				
No	Ref		Ref	
Yes	0.91 (0.52–1.58)		0.68 (0.41–1.13)	
SAPAS-trust other people				
Yes	Ref		Ref	
No	1.06 (0.59–1.88)		1.10 (0.67–1.82)	
SAPAS-lose temper easily				
No	Ref		Ref	
Yes	1.27 (0.68–2.39)		1.14 (0.66–1.95)	
SAPAS-an impulsive person				
No	Ref		Ref	
Yes	0.88 (0.57–1.37)		1.08 (0.75–1.55)	
SAPAS-a worrier				
No	Ref		Ref	
Yes	0.83 (0.54–1.29)		0.94 (0.64–1.37)	
SAPAS-depend lot on others				
No	Ref		Ref	
Yes	1.01 (0.59–1.72)		0.96 (0.61–1.50)	
SAPAS-a perfectionist				
No	Ref		Ref	
Yes	0.89 (0.60–1.34)		0.94 (0.67–1.34)	
SAPAS total (≥ 3)				
SAPAS total < 3	Ref		Ref	
SAPAS total ≥ 3	0.87 (0.57–1.34)		0.95 (0.65–1.37)	
Work-related variables				
On sick leave from unemployment or employment at randomization				
Unemployed	Ref		Ref	Ref
Employed	2.14 (1.24–3.71)**	2.14 (1.23–3.73)**	1.84 (1.19–2.83)**	1.81 (1.17–2.80)**
Socioeconomic status				
Salaried manager or self-employed	Ref		Ref	
Salaried worker	0.89 (0.54–1.46)		0.91 (0.58–1.40)	
Income transfer	0.69 (0.29–1.65)		0.89 (0.44–1.78)	
In education	0.67 (0.42–1.08)		0.75 (0.50–1.13)	
Sick leave duration at randomization				
Sick leave duration at randomization^3^	0.97 (0.92–1.02)		0.97 (0.93–1.01)	

Cox proportional hazards model with HR < 1 indicating a longer time to return to work and HR > 1 indicating a shorter return to work

*Significant at < 0.05

**Significant at < 0.01

***Significant at < 0.001

^1^10-year increase

^2^1-point increase

^3^1-week increase

**Table 5:** Demographic, health-related, psychological and work-related predictors for RTW in the depression group

Predictor variables	6 Months		12 Months	
	Univariate	Multivariate	Univariate	Multivariate
	HR (95% CI)	HR (95% CI)	HR (95% CI)	HR (95% CI)
Demographic variables				
Age^1^	1.18(1.01–1.37)*		1.14 (1.00–1.29)*	
Sex				
Female	Ref		Ref	
Male	0.96 (0.66–1.37)		0.82 (0.60–1.12)	
Marital status				
Not married	Ref		Ref	Ref
Married	1.33 (0.97–1.83)		1.61 (1.23–2.09)***	1.60 (1.22–2.09)***
Educational level				
Primary education only	Ref		Ref	
Secondary or vocational	1.07 (0.73–1.56)		0.99 (0.73–1.36)	
Professional or academic degree	1.49 (0.99–2.22)		1.40 (1.00–1.95)*	
Municipality				
Copenhagen	Ref		Ref	
Gentofte	1.44 (0.86–2.42)		1.72 (1.12–2.62)*	
Gladsaxe	1.13 (0.75–1.72)		1.21 (0.86–1.71)	
Lyngby	1.69 (1.04–2.75)*		1.72 (1.14–2.62)**	
Health-related variables				
Symptoms				
The Beck depression inventory^2^	0.98 (0.96–1.00)*		0.99 (0.98–1.00)	
Becks anxiety inventory^2^	0.98 (0.97–1.00)*		0.99 (0.97–1.00)	
Cohen’s perceived stress scale^2^	0.94 (0.92–0.97)***		0.97 (0.94–0.99)**	
The four-dimensional symptom questionnaire-anxiety^2^	0.96 (0.93–0.98)**		0.97 (0.95–1.00)*	
The four-dimensional symptom questionnaire-depression^2^	0.95 (0.91–1.00)*		0.96 (0.93–1.00)	
The four-dimensional symptom questionnaire-distress^2^	0.97 (0.95–1.00)		0.98 (0.96–1.01)	
The four-dimensional symptom questionnaire-somatization^2^	0.99 (0.96–1.01)		0.99 (0.97–1.01)	
The Karolinska exhaustion Scale^2^	0.99 (0.97–1.00)*		0.99 (0.98–1.00)	
Functioning				
The work and social adjustment scale^2^	0.96 (0.94–0.98)***		0.97 (0.95–0.99)**	
Diagnosis and comorbidity				
Diagnosis				
Anxiety	N/A		N/A	
Depression	N/A		N/A	
Stress	N/A		N/A	
Comorbidity				
No	Ref		Ref	
Yes	0.92 (0.66–1.28)		0.89 (0.67–1.17)	
Adult ADHD self-report scale (ASRS) ≥ 7 vs < 7				
ASRS < 7	Ref		Ref	
ASRS ≥ 7	0.76 (0.54–1.07)		0.81 (0.61–1.07)	
Psychological variables				
Quality of life				
Flanagans quality of life scale^2^	1.02 (1.01–1.03)***		1.01 (1.00–1.02)**	
Self-efficacy				
The illness perception questionnaire^2^	1.01 (0.97–1.06)		1.03 (0.99–1.07)	
Return-to-work self-efficacy^2^	1.08 (1.06–1.11)***	1.09 (1.06–1.11)***	1.06 (1.03–1.08)***	1.06 (1.04–1.08)***
The general self-efficacy scale^2^	1.03 (1.00–1.05)*		1.01 (0.99–1.03)	
Personality				
SAPAS-difficulty making/keeping friends				
No	Ref		Ref	
Yes	0.41 (0.22–0.74)**		0.70 (0.47–1.03)	
SAPAS-describe yourself as a loner				
No	Ref		Ref	
Yes	0.63 (0.39–1.03)		0.73 (0.50–1.07)	
SAPAS-trust other people				
Yes	Ref		Ref	
No	1.97 (1.11–3.49)*		1.55 (1.02–2.35)*	
SAPAS-lose temper easily				
No	Ref		Ref	
Yes	0.52 (0.24–1.12)		0.57 (0.32–1.02)	
SAPAS-an impulsive person				
No	Ref		Ref	
Yes	1.03 (0.73–1.46)		1.02 (0.76–1.36)	
SAPAS-a worrier				
No	Ref		Ref	
Yes	0.91 (0.65–1.27)		0.94 (0.72–1.24)	
SAPAS-depend lot on others				
No	Ref		Ref	
Yes	0.90 (0.54–1.52)		0.83 (0.53–1.29)	
SAPAS-a perfectionist				
No	Ref		Ref	
Yes	0.60 (0.43–0.83)**		0.65 (0.50–0.86)**	
SAPAS total (≥ 3)				
SAPAS total < 3	Ref		Ref	
SAPAS total ≥ 3	0.69 (0.49–0.97)*		0.77 (0.58–1.02)	
Work-related variables				
On sick leave from unemployment or employment at randomization				
Unemployment	Ref	Ref	Ref	Ref
Employment	3.14 (1.87–5.27)***	3.42 (2.03–5.78)***	2.27 (1.56–3.28)***	2.27 (1.56–3.31)***
Socioeconomic status				
Salaried manager or self-employed	Ref		Ref	
Salaried worker	0.68 (0.46–1.02)		0.83 (0.58–1.17)	
Income transfer	0.44 (0.24–0.82)**		0.63 (0.39–1.02)	
In education	0.66 (0.44–1.00)*		0.76 (0.53–1.09)	
Sick leave duration at randomization				
Sick leave duration at randomization^3^	0.99 (0.95–1.04)		0.99 (0.95–1.02)	

Cox proportional hazards model with HR < 1 indicating a longer time to return to work and HR > 1 indicating a shorter return to work

*Significant at < 0.05

**Significant at < 0.01

***Significant at < 0.001

^1^10-year increase

^2^1-point increase

^3^1-week increase

### Common mental disorders

#### Factors predicting slower RTW at 6 months

Results from the univariate analysis showed that slower RTW at 6 months was predicted by the following health-related factors: (a) diagnosis (depression was associated with slower RTW than anxiety disorders, and anxiety was associated with slower RTW than stress-related disorders); (b) psychiatric comorbidity; (c) lower levels of functioning and (d) symptom severity (higher scores on the anxiety, depression, stress and distress scales were associated with slower RTW at this time point). Slower RTW was also associated with the following psychological factors: (a) lower scores on both general (GSE), illness management (IPQ) and work-related self-efficacy (RTW-SE) measures; (b) lower QOLS scores; (c) certain personality traits (the SAPAS item having difficulties making and keeping friends) and (d) indication of personality disorder (SAPAS score > 3). Demographic factors predicting slower RTW at 6 months were (a) being unmarried; (b) younger age; (c) living in Copenhagen compared to the municipality of Lyngby and (d) educational level (having only primary education compared to secondary, vocational or academic education). Work-related factors associated with slower RTW included (a) being a student or a salaried worker compared to being a salaried manager or self-employed; (b) receiving transfer income and (c) being on sick leave from unemployment compared to employment.

Following the backward elimination algorithm, results from the multivariate analysis showed that slower RTW at 6 months was associated with lower scores on the RTW-SE and the QOLS; the SAPAS item having difficulties making and keeping friends; and being on sick leave from unemployment compared to employment.

#### Factors predicting slower RTW at 12 months

Findings from the univariate analysis showed that most health-related, psychological and work-related factors predicting slower RTW at 6 months also predicted slower RTW at 12 months. However, we found no association between neither age nor stress and slower RTW at 12 months. Moreover, having primary education was only associated with slower RTW compared to academic or professional training but not secondary education. Slower RTW at 12 months was also associated with being male, being unmarried and the SAPAS item would you describe yourself as a loner (Table 2).

The results of the multivariate analysis showed that slower RTW at 12 months was associated with being male, being unmarried, lower scores on QOLS and RTW-SE, psychiatric comorbidity and being on sick leave from unemployment compared to employment (Table 2).

In the CMD group, no association was found between RTW and ASRS score ≥ 7, sick leave duration or the rest of the SAPAS items.

### Stress

#### Factors predicting slower RTW at 6 months

In the stress group, univariate analyses showed that slower RTW at 6 months was associated with the following health-related factors: (a) higher scores on all symptom scales (except for the 4DSQ Anxiety); (b) lower levels of functioning and (c) psychiatric comorbidity. Slower RTW was also associated with psychological factors: (a) lower scores on QOLS, RTW-SE and GSE and (b) the SAPAS item having difficulties making and keeping friends. A significant demographic predictor was having primary education compared to secondary, vocational, professional or academic education. Finally, a significant work-related predictor was being on sick leave from unemployment compared to employment (Table 3).

In the multivariate analysis, only the work-related factor—being on sick leave from unemployment compared to employment—remained significant as a predictor of slower RTW at 6 months (Table 3).

#### Factors predicting slower RTW at 12 months

In the univariate analysis, most health-related, psychological, demographic and work-related factors predicting slower RTW at 6 months also predicted slower RTW at 12 months. Having only primary education compared to a professional or an academic degree predicted slower RTW but not compared to having secondary or vocational education. Slower RTW at 12 months was also predicted by the SAPAS item being an impulsive person and receiving transfer income compared to being a salaried manager or being self-employed (Table 3).

After backward elimination, the only predictors of slower RTW at 12 months were psychiatric comorbidity and being on sick leave from unemployment compared to unemployment (Table 3).

In the stress group, no association was found between RTW and sex, marital status, municipal affiliation, ASRS score ≥ 7, sick leave duration or the rest of the SAPAS items.

### Anxiety

#### Factors predicting slower RTW at 6 months

In the anxiety group, univariate analysis showed that slower RTW at 6 months was predicted by higher scores on the anxiety, distress and stress symptom scales; lower scores on QOLS and GSE; and being on sick leave from unemployment compared to employment (Table 4).

After backward selection, the multivariate analysis showed that slower RTW at 6 months was predicted by being on sick leave from unemployment compared to employment.

#### Factors predicting slower RTW at 12 months

In the univariate analysis, factors predicting slower RTW at 12 months were higher scores on the anxiety, distress and stress symptom scales, lower scores on QOLS, having primary education compared to a professional or an academic degree and being on sick leave from unemployment compared to employment (Table 4).

After backward selection, multivariate analyses showed that being on sick leave from unemployment compared to employment and lower QOLS scores predicted slower RTW at 12 months (Table 4).

No association was found in the anxiety group between RTW and age, sex, marital status, socioeconomic position, municipality affiliation, psychiatric comorbidity, ASRS score ≥ 7, sick leave duration, IPQ, RTW-SE or any of the SAPAS items.

### Depression

#### Factors predicting slower RTW at 6 months

In the depression group, the univariate analysis showed that the following health-related factors predicted slower RTW at 6 months: (a) symptom severity (higher scores on the depression, anxiety and stress symptom scales BDI II, BAI, PSS, 4DSQ Anxiety, 4DSQ Depression and KEDS) and (b) lower levels of functioning. Significant psychological factors included (a) lower scores on QOLS; (b) lower scores on GSE and RTW-SE; (c) the SAPAS items having difficulties in making and keeping friends and would you describe yourself as a perfectionist and do you trust other people (“no”) and (d) a SAPAS score ≥ 3, indicating the presence of a personality disorder. Demographic predictors included (a) younger age and (b) living in Copenhagen compared to the municipality of Lyngby. Significant work-related predictors were (a) receiving transfer income or being a student compared to being a salaried manager or self-employed and (b) being on sick leave from unemployment compared to employment (Table 5).

After running the backward elimination algorithm, the multivariate analysis showed that lower levels of RTW-SE and being on sick leave from unemployment compared to unemployment were significant predictors for slower RTW at 6 months (Table 5).

No association was found between RTW and sex, education, marital status, sick leave duration, ASRS score ≥ 7 or IPQ at 6 months (Table 5).

#### Factors predicting slower RTW at 12 months

At 12 months, findings from the univariate analysis showed that health-related factors predicting slower RTW included (a) higher symptom score on the PSS scale and the 4DSQ Anxiety scale and (b) lower levels of functioning. Significant psychological predictors were (a) lower levels of QOLS; (b) lower levels of RTW-SE and (c) the SAPAS item would you describe yourself as a perfectionist and do you trust other people (“no”). Significant demographic predictors were (a) younger age; (b) living in Copenhagen, compared to the municipalities of Gentofte or Lyngby; (c) being unmarried and (d) having primary education compared to a professional or an academic degree. A significant work-related factor was being on sick leave from unemployment compared to employment (Table 5).

After running the backward elimination algorithm, multivariate analysis showed that slower RTW at 12 months was predicted by lower levels of RTW-SE, being on sick leave from unemployment compared to employment and being unmarried.

At 12 months, no association was found between RTW and sex, psychiatric comorbidity, ASRS score ≥ 7, IPQ and GSE.

## Discussion

In this study, we investigated factors predicting slower RTW in people on sick leave with depression, anxiety disorders and stress-related disorders; similarities and differences between the CMD group and the subgroups and between the subgroups; and the question of whether factors predicting RTW changed from 6 to 12 months in each of the subgroups and in the CMD group as a whole.

### Similarities and differences in RTW predictors across time points and diagnoses

Findings from the multivariate analyses revealed not mainly similarities but also a few differences between the CMD group and the subgroups and between the subgroups. Lower return-to-work self-efficacy was identified as a factor predicting slower RTW at 6 and 12 months in the CMD group and in the depression subgroup. Poorer quality of life was a predictor for slower RTW in the CMD group at both time points and in the anxiety subgroup at 12 months. Psychiatric comorbidity predicted slower RTW at 12 months in the stress subgroup. Being male and being unmarried were significant demographic predictors for slower RTW in the CMD group and in the depression subgroup, but only at 12 months. The most prominent factor predicting slower RTW was being on sick leave from unemployment compared to employment. This factor was significant at 6 and 12 months both in the CMD group and in the stress, anxiety and depression subgroups.

In the univariate analysis, most factors predicting RTW at 6 months also predicted RTW at 12 months. A higher symptom burden at baseline reduced the likelihood of RTW at both time points in the CMD group and in all diagnostic subgroups. These results are to some extent consistent with the systematic review by Fisker et al. (2022), but other studies (Hellström et al. 2018; Vemer et al. 2013) have not found an association between symptom severity and RTW. The conflicting results might indicate that higher levels of symptoms alone do not predict RTW likelihood or the pace of the RTW process, but that the impact on RTW is mediated by environmental factors, such as opportunities for workplace adaptations and support (Corrigan 2001; Fyhn et al. 2021). In the stress subgroup, psychiatric comorbidity was associated with slower RTW at 12 months. Most psychiatric comorbidities in the stress subgroup consisted of mild depression, and one may argue that the participants should instead have been included in this study with a depression diagnosis, as defined by the WHO ICD-10. We therefore emphasize that this finding should be interpreted with caution. Many of the demographic factors predicting slower RTW at 6 and 12 months (being male, being unmarried, low education and low income) are also consistent with findings in other studies (Ervasti et al. 2017; Fisker et al. 2022; Lagerveld et al. 2010b). However, contrary to studies (Ervasti et al. 2017; Fisker et al. 2022; Hellström et al. 2018) that showed an association between older age and decreased likelihood of RTW, we found that younger age predicted slower RTW in the CMD group at 6 months and in the depression subgroup at 6 and 12 months. These findings may point to the existence of a vulnerable group among people on sick leave with depression, the NEET group, that requires tailored vocational rehabilitation interventions. NEET—an acronym for “Not in Employment, Education or Training”—is an internationally consolidated indicator used to describe school-to-work transition difficulties in a vulnerable population of youth at risk of marginalization and social exclusion. A systematic review from 2021 found that NEET status was associated with mood disorders (OR 1.43, 95% CI 1.21–1.70) and other mental health conditions. (Gariépy et al. 2021) While researchers have discussed whether younger age is associated with greater flexibility and readiness to change, and therefore with faster RTW (Hellström et al. 2018), findings from our study suggest that a subset of young people are less likely to return to work and therefore risk long-term disadvantages. Hence, customizing vocational rehabilitation interventions to target this group seems warranted.

An interesting finding in our study is that psychological predictors of RTW differed between the anxiety and depression group. In the depression group, slower RTW was predicted by RTW-SE at both time pints, while RTW-SE was not a predictor for RTW in the anxiety group at any time point, neither in the univariate nor in the multivariate analysis. Instead, RTW was predicted by QOL at both time points. At 12 months, the association between QOL and RTW remained significant in the multivariate analysis.

Self-efficacy can be understood as an individual’s confidence in his or her ability to perform certain behaviours effectively. In self-efficacy theories, experiences of mastery is connected to specific demand areas (Bandura and Adams 1977). Thus, RTW-SE refers to the person’s self-efficacy in the RTW process and is commonly targeted in vocational rehabilitation interventions, for instance, work-focused CBT. In our study, lower QOL predicted slower RTW at 6 and 12 months in the anxiety group, which suggest that there might be other areas in a person`s life than work that also require attention in vocational rehabilitation interventions.

Being on sick leave from unemployment compared to employment was a prominent factor predicting slower RTW at 6 and 12 months in the CMD group and the stress, anxiety and depression subgroups. This result aligns with findings from other studies (Audhoe et al. 2012; Farrants et al. 2018; Lammerts et al. 2016; Netterstrøm et al. 2015; Virtanen et al. 2011). One may conclude that core components deemed to be active ingredients in effective RTW interventions, such as work-focused CBT and workplace change (Mikkelsen and Rosholm 2018; Nieuwenhuijsen et al. 2020), do not apply to people on sick leave from unemployment. Our findings highlight the need to develop job search support and labour marked access as core components in vocational rehabilitation intervention. However, “conventional” supported employment interventions, like IPS, which are oriented towards obtaining ordinary employment or education, have not demonstrated effectiveness in people with CMD (de Winter et al. 2022; Hellström et al. 2021). Therefore, more research is needed on how to best support RTW processes among people with CMD on sick leave from unemployment. It has been proposed that demand-side employment research can play a valuable role in advancing vocational rehabilitation interventions (Chan et al. 2010). Demand-side employment research goes beyond individualistic models that emphasize building capacities and stamina as key ingredients in RTW interventions, focusing instead on factors, such as employer demands, organizational behaviours and labour economy as determinants of vocational outcomes (Chan et al. 2010; Delman et al. 2017). A scoping review on demand-side employment interventions for people with CMD identified only six studies, all of which included employed participants (Bauer and Gewurtz 2022). More research is needed in this area, for instance, on how attitudes and stigma, financial incentives, workplace accommodations and options for support can impact hiring practices and increase job retention and employment for people with CMD on sick leave from unemployment.

### Clinical and scientific implications

Findings from our study can guide clinicians delivering vocational rehabilitation interventions to people on sick leave with depression, anxiety disorders and stress-related disorders. Return-to-work self-efficacy and quality of life are relevant areas to address but may impact the RTW process differently depending on whether the individual is on sick leave with depression or anxiety disorders. The findings also suggest that a heavier symptom burden leads to slower RTW in the short and the long term, but appropriate support may moderate this association.

Our study has also identified subgroups within the target group that highlight the need to evaluate and further develop vocational rehabilitation interventions. People on sick leave from unemployment have needs that are not addressed by current vocational rehabilitation interventions. Demand-side employment research may produce knowledge about strategies to improve vocational outcomes for this group. Findings also indicate that younger age reduces the likelihood of RTW in people on sick leave with depression. Unemployment or interrupted education entails a significant risk of long-term adverse consequences, and more research is needed on how to customize effective vocational rehabilitation interventions to younger people living with depression.

### Strengths and limitations

To the authors’ knowledge, this is the first study investigating predictors of RTW in people on sick leave due to CMD (both within the CMD group as a whole and within the stress, anxiety and depression subgroups) that has applied self-reported measurement and clinical assessment, including information about health-related and psychological factors. This study includes participants on sick leave from employment and from unemployment, thus ensuring diverse representation of important demographic, social and clinical characteristics in the population. The register-based data ensured information on RTW for all participants in this study at 6 and 12 months follow-up. All these factors strengthen the study findings.

However, this study has certain limitations that need to be addressed. First, the data stem from participants in two RCTs. Thus, we cannot assume that this population is representative of the general population of people on sick leave with CMD. Moreover, the fact that two-thirds of the participants received an experimental intervention may mean that results from this study cannot easily be transferred to contexts beyond the IBBIS project. We addressed this matter by adjusting for intervention group in the analysis. Another option was to only analyse data from participants included in the control group in the IBBIS project, but doing so would have resulted in markedly less power. Analyses in the subgroups were based on samples that differed in size, for instance, between 636 participants in the stress group and 222 participants in the anxiety group. These differences could have influenced the power within each subgroup to identify predictors and subsequently affect the reliability of comparing predictors across subgroups. Missing data affected a minor proportion of baseline measures. To account for this, a multiple imputation strategy was carried out. Predictable missingness structures were thus accounted for, and since no data are missing in the outcome measures, the risk of bias is small.

Univariate analysis showed that many variables were significant individually, but fewer variables were found significant in the overall predictive model, probably because many of the psychometric variables are highly correlated. A correlation matrix (supplementary material) showed that most correlations to be around 0.3 and 0.7. The factors with high correlation coefficients often reflected the same underlying feature, e.g. anxiety as measured with BAI and the 4DSQ Anxiety.

### Conclusion

The aim of this study was to investigate predictors of RTW in people on sick leave with CMD at 6 and 12 months and across the subgroups diagnosed with depression, anxiety disorders and stress-related disorders. While the univariate analysis showed that symptom burden impacted RTW at both time points and across diagnosis, the multivariate analysis showed that RTW-SE predicted RTW in the CMD and depression groups but not in stress or anxiety disorder groups. In the anxiety group, QOL predicted RTW at 12 months. The results highlighted that people on sick leave with CMD from unemployment may constitute a vulnerable group that requires adapted vocational rehabilitation interventions. Moreover, further studies are needed to gain more knowledge on how environmental factors related to the workplace influence RTW.

## Supplementary Information

Below is the link to the electronic supplementary material.

Supplementary file1 (DOCX 101 KB)

## Data Availability

Data sharing is not permissible due to the EU General Data Protection Regulation 2016/679 (GPDR).
