# Supplementary figures and images for: Predictors of return to work for people on sick leave with depression, anxiety and stress: secondary analysis from a randomized controlled trial
Source: Int Arch Occup Environ Health. 2023 Mar 18;96(5):715–34. doi: 10.1007/s00420-023-01968-7 (PMC10220117; doi:10.1007/s00420-023-01968-7)

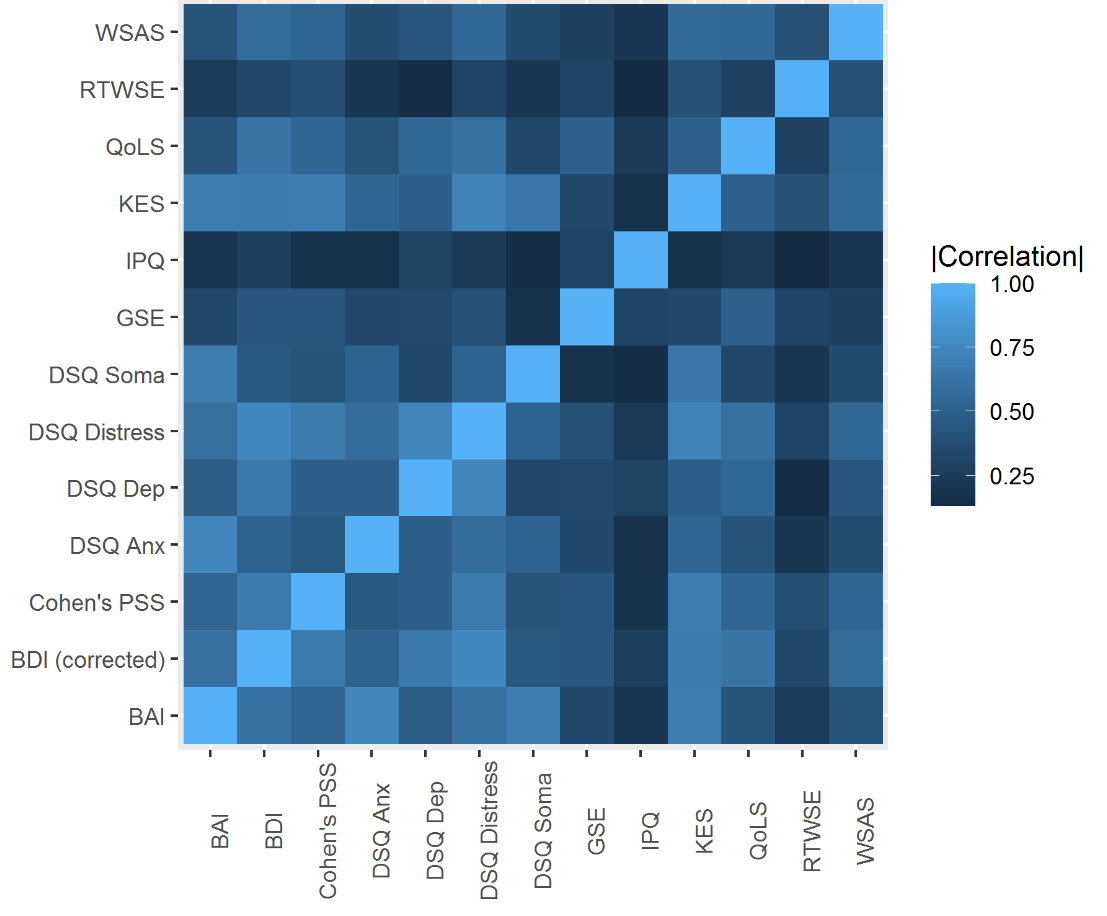


Figure 1: Results from multicollinearity analysis.

Supplement: Supplementary file 1 — Supplementary file1 (DOCX 101 KB) [file 420_2023_1968_MOESM1_ESM.docx]
